# Popliteal Aneurysm

**Published:** 1890-04-19

**Authors:** 


### POPLITEAL ANEURYSM.

A clinical lecture on "Aneurysm" has been delivered by Mr. Christopher Heath. A case is taken in illustration. A female paralysed on one side developed aneurysm of the popliteal artery on the other side, arising, as the lecturer observed, from the greater strain the artery of this limb was subjected to. As a rule, popliteal aneurysm occurs to males, who are subjected to more strain of the part than females. The patient complained of a lump in the popliteal space. But it was evidently aneurysmal, because it pulsated, and compression of the femoral artery stopped the pulsation. Still, the lecturer remarked, every pulsating swelling is not an aneurysm. But on pressing the swelling while the femoral artery was commanded, the swelling was diminished in bulk. There was also a bruit, but even a bruit is not proof of an aneurysm. The first treatment tried was digital compression, the object being to stagnate the blood in the aneurysm. But pressure kept up for four hours by relays of dressers on the femoral artery was not followed by improvement. Then flexing the limb was tried, and forcible flexure maintained all one day produced no apparent result. So instrumental pressure was had recourse to. This was effected by a Cartes' torniquet to the groin, and a horse-shoe torniquet below. The pressure, however, was not complete, for cure was hoped for by laminated fibre deposits, and not by clot. But this treatment did not succeed, although the patient had been prepared by a diet composed chiefly of meal and little fluid, and by the exhibition of iodide of potassium. Therefore the femoral artery was eventually ligatured. For ligature of an artery Mr. Heath prefers good, strong aseptic silk, mentioning that catgut may slip or soften. Mr. Heath also ties the ligature tightly, which some do not advise. In the case in question the application of Esmarch's bandage was not deemed advisable, for there is some risk of gangrene. Mr. Heath does not approve of injections, or the introduction of horse-hair or of thin wire, or of the injection of ergotine into the neighbourhood, or of forcible manipulation (with the view of disturbing a clot and blocking the artery), all of which have been proposed as a treatment for aneurysm. Another case of popliteal aneurysm is reported by Fleet Surgeon J. M. Stone, R.N. (*British Medical Journal*). There was a pulsating tumour in the left popliteal space, with a distinct bruit. Digital pressure of the femoral was employed for twenty-four hours, Signoroni's torniquet being then substituted for a couple of hours, when digital pressure was again resorted to for twenty hours. Considerable œdema of the leg and thigh resulted, with little diminution of pulsation. On the subsidence of the œdema the torniquet was again used with negative result. Having regard to the size of the tumour, and to the presence of a loud bruit, Esmarch's bandage was not considered advisable, so the femoral was ligatured at Scarpa's triangle, kangaroo tendon was used, the incision closed by carbolised gut sutures, and dressed antiseptically. In one month the patient was discharged to duty. The feature of interest connected with this case appears to turn on the fact that the protracted course of pressure seems to have had a favourable influence in initiating collateral circulation before deligation was performed, an inference which the total absence of œdema, with trifling pain and inconvenience after the application of the ligature seems to justify. The reporter adds, "closure of the wound by first intention, and the total disappearance of ligature and sutures may be worthy of note."

